# Optimising a co-production framework for developing public health interventions: application and testing of school-based Research Action Groups

**DOI:** 10.1186/s12961-023-01086-3

**Published:** 2023-12-14

**Authors:** Hayley Reed, Simon Murphy, Rhiannon Evans

**Affiliations:** DECIPHer, School of Social Sciences, Cardiff University, Spark, Maindy Rd, Cardiff, CF24 4HQ United Kingdom

**Keywords:** Co-production, Intervention development, School health, Mental health and wellbeing, Process evaluation

## Abstract

Existing frameworks for developing public health interventions have limited guidance for researchers on how to work with stakeholders to co-produce context-specific interventions. To address this, a promising framework for the co-production of public health interventions was developed through a previous review of school-based co-production. The present study aimed to optimise this co-production framework through applying and testing it in real-world secondary school contexts. Within schools, Research Action Groups (RAGs) were established with multiple school stakeholders supported by an external facilitator to use school data to produce school-specific mental health and wellbeing interventions. A mixed method process evaluation of two contextually diverse case study secondary schools in the UK was used to optimise the co-production framework. The process evaluation sought the views of those involved (students, school staff, school Senior Management Team (SMT) members, and the external co-production facilitator/researcher) on the co-production functions they were involved in so they could be modified, if needed. Data collected for the process evaluation during co-production were a researcher diary (*n* = 45 entries), and observations of student photography (*n* = 21) and RAG meetings (*n* = 8). Post co-production, interviews, and surveys with RAG students (*n* = 18) and staff (*n* = 8), and two school-specific SMT focus groups (*n* = 10) were conducted. The study identified four recommendations to optimise and integrate co-production into real world practice. They include the need to: assess schools for their readiness to undertake co-production; more effectively communicate the necessity to have stakeholders from the whole system involved; work with SMTs throughout co-production functions; involve stakeholders outside the school to support producing solutions to change school mental health and wellbeing priorities. The framework is intended to be used by researchers to integrate stakeholders into a shared decision-making process to develop interventions that meet the needs and contexts of individual schools. It could be transferred to other settings to support the development of public health interventions for other health areas, and populations. Further evaluation to test its use in other settings is needed.

**Supplementary Information:**

The online version contains supplementary material available at 10.1186/s12961-023-01086-3.

## Background

The effectiveness of complex health interventions is affected by how interventions interact with their context during implementation [4, 38]. For example, variability in effectiveness has been found for mental health school-based interventions, which are based on health education [20], and on multicomponent interventions focused on Social and Emotional Learning (SEL) [8] and Health Promoting Schools [22]. Programme evaluations have found that variations in implementation are partially due to a lack of fit between the intervention and school contexts [10, 11, 19]. For example, Durlak’s [10] narrative review of SEL programmes found two important factors affecting implementation were the compatibility of the programme with the school’s priorities and values, and how relevant the school delivery staff thought the programme was to address the schools’ needs.

To address this, overarching and phase-specific intervention guidance increasingly emphasises accounting for context [9, 28, 34]. The Medical Research Council’s [9] guidance on context outlines key features that can be used to understand how interventions are developed and implemented, and how their effects can vary. First, context is defined broadly, including multiple socio-ecological aspects from the characteristics that vary between individuals to the environmental characteristics that affect the whole population. However, not all of these aspects of context are relevant to understanding how every intervention works. Secondly, interventions are social entities implemented and experienced by people, who themselves are influenced by their social, cultural, historical, and political environments. Lastly, as some interventions intend to change context, interventions can be viewed as ‘events in systems’ [17]. Complex systems perspectives are advocated as one approach to understand how an intervention and its context interact [17, 26, 31]. This views schools as complex adaptive systems [21] that either accept and adapt to interventions or strive to maintain order and reject it [17].

Whilst a number of intervention development approaches have been identified [28], co-production is endorsed to allow the target population to voice their understandings of how an intervention will work within a given context [9, 26]. Stakeholders can co-develop programme theory [34] which it is believed will ensure limited health improvement resources are targeted towards the correct mechanisms [25]. They can also indicate early in development processes what could enable and hinder implementation [14].

There are two main issues with the current co-production literature that are relevant to public health intervention development. First, there are numerous definitions of co-production as it is a complex, contested construct that can involve multiple stakeholders in a multitude of ways [42]. This makes it difficult for researchers to understand what co-production is and what differentiates it from other forms of participatory practice. For the current project, co-production is understood as the involvement of stakeholders in shared decision-making processes to create knowledge on interventions. This should include stakeholders from multiple levels within complex systems [26] especially those that are traditionally excluded from these decisions-making processes [42], for example, the target population. To differentiate co-production from other participatory practice, the central decision-making functions of problem-setting and problem-solving have been used as a threshold to classify it [2, 15, 30]. This ensures that stakeholders are involved in developing an understanding of the health problems and theorising how these can be changed.

Secondly, existing guidance and frameworks for developing public health interventions do not focus on how researchers can co-produce with stakeholders in real world settings. Much of what is produced about co-producing interventions is rhetoric; encouraging researchers to co-produce without examples of how to do this. Notable exceptions are Six Steps in Quality Intervention Development [41] and Hawkins et al.'s [18] Co-production and Prototyping Framework. However, these focus on co-producing standardised content and delivery processes for use in many different settings e.g. in multiple schools. While this is a plausible resolution to implementation problems, evidence shows that allowing schools to tailor interventions to their own needs and settings can also enable implementation [32]. From here on, this will be known as context specific co-production as co-production is run within a setting to produce health interventions for that setting only.

### Project aim and development of the initial framework

**Fig. 1:**
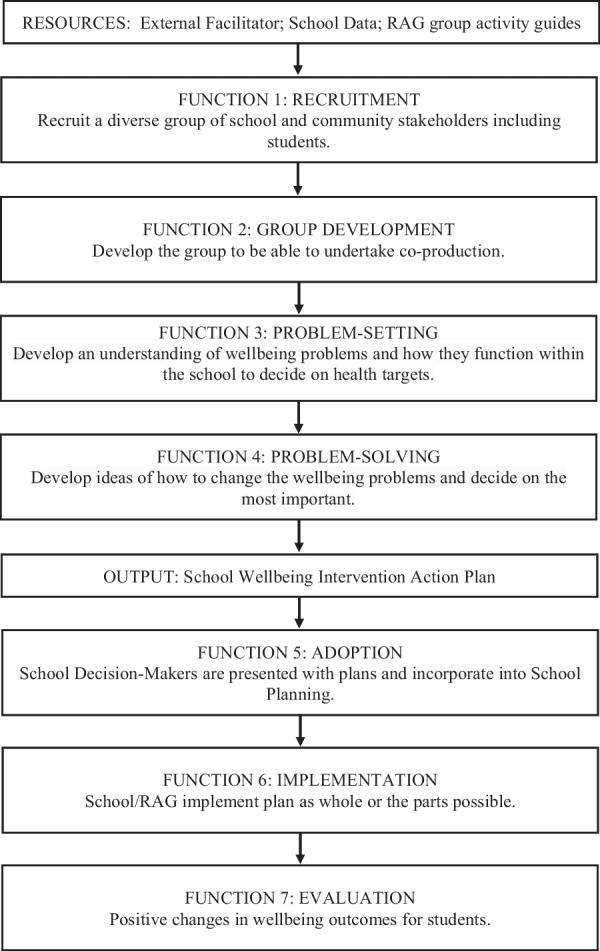
Initial Framework for Co-producing Public Health Interventions

The aim of the present study was to optimise a context-specific co-production framework through applying and testing it in real-world secondary school contexts. An initial framework was established through a review of secondary school studies focused on co-producing context-specific health interventions [30]. The review found that co-production forms varied, for example, in how capacity was built to support co-production. The review also outlined the shared functions (purposes) projects had; these shared functions were used to develop the initial framework shown in Fig. 1.

It was decided that the emergent framework needed further testing to optimise it. Research Action Groups (RAGs) were chosen to test the framework. RAGs are school-level decision-making bodies that involve multiple stakeholders which can include school staff, students and sometimes Governors and parents [36]. They were chosen to test the framework for two reasons. First, the previous review showed they were a popular approach to school-based health co-production and they have continued to be (e.g. [36]). Second, the review found some evidence of these structures being empowering and acceptable to the stakeholders involved [1], feasible to implement within secondary schools (e.g. [2]), and a suitable structure to achieve shared decision-making for health intervention development [5, 12]. The current study attempted to build on the knowledge base already developed by the review.

### Research action groups

Schools were asked to establish RAGs with multiple stakeholders including a member of their SMT and nominate a link teacher responsible for delivering co-production. To inform RAGs of their school’s mental health and wellbeing context, the RAG students undertook a photography project with the facilitator to elicit their understandings of mental health and wellbeing, and school student health survey results were obtained. Subsequently, the RAGs met at least four times to use these data and were provided with proposed activities to share decision-making on problem-setting and solving. The emerging mental health and wellbeing issues and solutions formed school-level intervention plans.

During the co-production functions, the external facilitator promoted the preferred forms for functions to link teachers, although they were allowed to tailor them to their own context. For example, schools were guided to recruit students through assemblies (preferred form), so all young people had an equal chance to be involved. In both schools, assemblies were used but staff also approached certain students to increase the diversity of the RAGs. The direction given to schools about preferred forms was developed through the review of 22 studies on school-based co-production [30] and in discussion with a young people’s advisory group who advised on what was the most appropriate forms from the perspective of secondary school students. The main aim was to give schools’ guidance rather than be prescriptive on the forms they used, however (as shown in Additional file 1: Table S3) there were instances when schools needed more support from the facilitator to achieve the purposes of co-production.

## Methods

### Study theoretical approach

A complex system perspective was adopted as the importance of context in intervention research has been increasingly recognised [17, 26, 31]. As described above, this frames interventions as ‘events in systems’ [17] and recognises schools as Complex Adaptive Systems [21, 26]. Within this approach, there is a distinction between intervention functions (the intervention purposes) and forms (the strategies used to meet each function) [29]. So the study focused on how the introduction of co-production functions disrupted the contexts they were delivered in, and how the school systems and its agents responded.

### Study design

The study used a mixed method process evaluation with two contextually diverse Welsh case study secondary schools to optimise the co-production framework. The process evaluation sought the views of those involved in the co-production functions (students, school staff, school SMT members, and the external co-production facilitator/researcher). It focused on collecting data in the four domains of implementation, context, decision-making mechanisms and social validity.

The first three domains were taken from process evaluation guidance [24]. The domain of context was considered as crosscutting and focused on understanding how school contextual factors supported or hindered co-production functions, shaped participants reactions to functions, and whether these hindered or facilitated the production of intervention plans. Implementation was the extent to which co-production functions were delivered and how this delivery was achieved. Assessing decision-making mechanisms focused on whether the hypothesised causal pathways to change were triggered, how they worked, and how participants’ actions and contextual circumstances affected them. Social validity was used in substitute of evaluating outcomes as this study followed up the RAGs from recruitment of members until adoption processes only (see Fig. 1, Functions 1 to 5). Social validity was the extent to which the co-produced intervention plans were perceived as relevant and implementable in the schools they were created.

This paper focuses on co-production functions and draws predominately on qualitative data linked to the domains of implementation and context. A further paper exploring the mechanisms of shared decision-making will follow to complement this paper.

### Case studies

A case study methodology in two schools was used to allow an in-depth, idiographic inquiry to understand how stakeholders’ experienced the co-production functions, and hence, what modifications could be made to optimise the framework for future studies. The two case study schools were purposively sampled based on their variations in entitlement to Free School Meals (FSM), school size, and geographical location. School 1 was a low FSM (below national average), high population (above national average) school located in an urban area. School 2 was a high FSM (above national average), low population (below national average) school located in a semi-rural area.

In school 1, the RAG was made up of 10 students aged 11–18 years old, and six staff. They were the link teacher who was the lead for Inclusion and Wellbeing and a SMT member, two Wellbeing Directors for Key Stages 3 and 4, two Wellbeing Officers and a Youth Mentor who worked with disengaged students in the school. However, while two Wellbeing Directors signed up for the RAG neither attended a meeting. In school 2, 12 students aged 11–18 years old were recruited and were accompanied by four staff in terms of the lead for wellbeing (SMT member) and their deputy (the link teacher and another SMT member), the Key Stage 4 Wellbeing and Attendance Manager and Youth Engagement Officer who provided informal support for all students.

### Data sources

Table 1 gives an overview of all data sources collected, and the type and numbers of participants that data was collected from for each data source. Data sources included:

1. Electronic Research Diary—was kept with 45 individual day entry’s where both schools were visited on some days. The researcher documented every contact with or visit to a school. Draft entries were created in schools and expanded on and formalised later in the day.
2. Semi-Structured Individual/Paired observations of photography—of students undertaking their photography work and their elicitation sessions (*n* = 21 observations, *n* = 22 students). Sessions were audio recorded because the researcher’s dual role of facilitator and evaluation researcher. The researcher made preliminary notes during observations but used recordings (within 48 h) to develop comprehensive observations.
3. Semi-Structured Group Observations—Four observations of RAG meetings were undertaken in each school (*n* = 8). These were audio recorded offering the researcher/ facilitator the opportunity to develop more comprehensive observations post-meetings.
4. RAG Survey—Post co-production, RAG members were issued with a self-completion survey about their co-production experience, with 26 returned surveys from 18 students and eight staff. This survey expanded on one used in another study [12].
5. Semi-Structured RAG Interviews—Individual interviews were undertaken post co-production with 18 students and eight RAG staff. The researcher also re-visited participants’ survey answers so they could provide clarity where needed.
6. Semi-Structured Focus Groups—Two focus groups with 10 (school 1 = 4; school 2 = 6) SMT members were conducted post-co-production. There was crossover between RAG and SMT membership in both schools.

**Table 1:** Data collected for the process evaluation

Data source	Informant	School 1 N (%)	School 2 N (%)
Research diary	Researcher	32-pages with 45-day entries	
Individual observation sheets	Researcher	10 (100)	11^a^ (100)
Group observation sheets	Researcher	4 (100)	4 (100)
Student RAG member survey	Student RAG member	7 (70)	11^a^ (100)
Staff RAG member survey	Staff RAG member	4 (67)	4 (100)
Student interviews	Student RAG member	7 (70)	11^a^ (100)
RAG staff interviews	Staff RAG member	4^b^ (67)	4^b^ (100)
SMT focus group	SMT	4^b^ (80)	6^b^ (86%)

^a^Two students asked to conduct data collection as a pair so actual number of students is one higher

^b^Some staff were members of both the RAG and the SMT so featured in both data collection methods

Interviews and focus groups were audio recorded and transcribed by a transcription service.

### Data analysis

Three steps were used to analyse the dataset which identified four recommendations to optimise the framework (see results sections).

- Analysing Raw Data: Quantitative data from observations and surveys were analysed into means, data ranges and standard deviations within Excel. Attendance data for meetings was also tabulated. For qualitative data, ‘Codebook’ thematic analysis [6] was used to order and synthesise the dataset in NVivo 12. The first author deductively coded data into a priori themes based on the process evaluation domains. The data was then inductively coded into subthemes based on reflective engagement with the dataset through reading and re-reading all themes, and constantly collapsing and refining subthemes. Draft memos were constructed and shared with team members for discussion and refinement.
- Synthesising Quantitative and Qualitative Data: A second memo was created amalgamating quantitative data with qualitative memo summaries to explain each other [24]. For example, stakeholders’ views on co-production functions from interviews were used to understand the quantitative survey ratings given for the usefulness of functions (see Additional file 1: Table S2).
- Integrating Findings with the Wider Literature: A final memo described if and how the functions of the initial framework (Fig. 1) should be modified. It achieved this through drawing on the findings in step b and the research literature linked to school-based co-production studies and complex systems thinking.

## Results

**Fig. 2:**
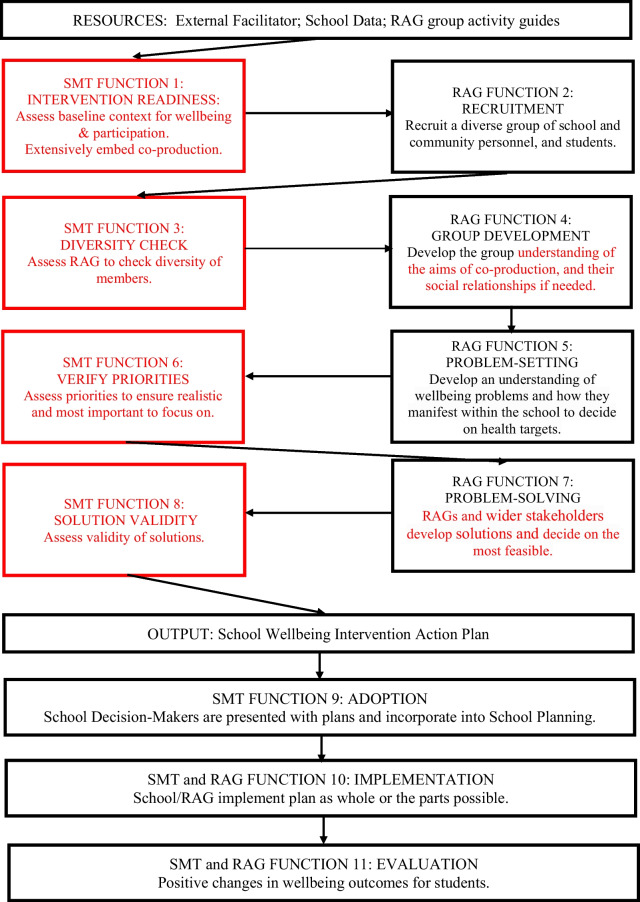
Optimised Framework for Co-producing Public Health Interventions

Previous review findings [30] were used to develop a promising co-production framework (see Fig. 1). Overall, co-production functions were well received and deemed ‘very useful’ or ‘quite useful’ by school stakeholders (Additional file 1: Table S2). As the aim of the current study was to optimise the framework, the results section focuses on reporting four recommendations used to do this, in response to testing it in a real-world setting. Each recommendation has a statement explaining the change to the initial framework (see also Fig. 2, red text).

### Assessing intervention readiness

The first recommendation introduced a new function termed ‘Intervention Readiness’ to assess the alignment of baseline school contexts with the tenets of co-production (in terms of involving stakeholders and addressing mental health and wellbeing). This should allow schools to understand their starting systems, specifically the structures and processes already in place for student wellbeing and involvement in decision-making. This was needed because the two schools used different strategies to embed co-production termed intensiveness and extensiveness. This showed that schools’ baseline readiness to implement co-production will differ and needs to be addressed before introducing co-production.

In school 1, co-production was embedded through an intensiveness as the link teacher deeply engaged in all planning and implementation of activities. Her activist approach led to a rapid start and her drive facilitated some functions such as recruiting students. However, this centralised control was a barrier to other functions such as other staff not passing on messages to recruited students to attend meetings.

This paralleled the lack of focus on student wellbeing from the wider staff team. This was demonstrated by the two Directors of Key Stage 3 and 4, who were responsible for student wellbeing, not attending a co-production meeting even though meetings were planned to accommodate them. Their non-attendance was noticed by students and other staff:

RES: yeah, but he was too busy for us. I don’t think he could be bothered to come. Because when I went to see him, he was really, he was really, he wasn’t very nice.

INT: Oh, because you went to ask him [to attend meeting] didn’t you?

RES: I asked him to come, but he was like, he wasn’t the nicest person about it, I said “Oh we were wondering if you could come?”, he was like “I’m busy, so I can’t, sorry”, like that was just like, oh you could just speak a bit nicely…

INT: Yeah, but you don’t think (KS4WD), was interested?

RES: I think he was, but I just didn’t think he, I think he, he has priorities. Even it was about what his job wellbeing, but he didn’t seem to really care that he missed, because he didn’t even show up to the other meetings (School 1, Student 7).

Also, there was a lack of intervention understanding and engagement from the rest of the SMT. This was shown when the researcher met with them, as they did not understand the project, had not read the resultant intervention plan, and constantly referred questions to the Link Teacher to answer. This was attributed by stakeholders to the intervention coupling with a baseline context where the Link Teacher was solely responsible for student wellbeing. Resultingly, the SMT did not commit to any actions in the wellbeing plan in school 1.

Whereas in school 2, the embedding of the intervention was characterised by an extensiveness throughout the school subsystems. For example, the Headteacher was onboarded before the school committed to take part, and they strongly supported the implementation of co-production throughout. This increased the system readiness to consider the RAG’s plan which resulted in the SMT committing to three actions at project end.

Further, all staff were informed of the project through a staff meeting allowing it to permeate even further into the context and making teaching staff aware of their duty to release students from lessons for co-production activities. This was accompanied by a diffusion of intervention responsibility which improved intervention success. From initiation, co-production implementation was supported by other members of the wellbeing team. This shared responsibility typified the routine work format of the wellbeing structures:

RES: Now there’s a lot of dovetailing going on there and there’s a lot of grey area about who’s in charge of what… but we do tend to work together. I’m, my role really should be just the PSE part of it and how the kids are dealt, er, are taught that within lesson time or curriculum time …

INT: Okay.

RES: … then the whole school like anti-bullying and anti-smoking should really be done through the whole school which is (other staff name) but then as part of me being on SMT this year, I sort of said, well I’ll help out with that, so we sort of decided to sort of do it all together. (School 2, Staff 1).

The co-production intervention also coupled well with school 2’s baseline context which was one of students feeling cared about, safe, and able to help seek if needed:

RES: Yeah, ‘cos like our teachers really care about us. If they see us, if they see us like not normal, like I was looking angry, upset or not like “right”, they’ll just immediately talk to you, without, without thinking about it, they’ll just click, like a click of the finger, they’ll talk to you and it’s really good for that, ‘cos like students get more friendship with teachers doing that. Talking about the problems in their life with teachers and like support teachers …it really helps them. (School 2, Student 6).

These differences in embedding and coupling with the baseline context highlight the need to assess the readiness of individual schools to receive co-production [17]. This should take the form of auditing the context and substructures already linked to the public health topic which co-production targets. This can then be addressed, where needed, through further work to harness appropriate staff activities to inform and onboard actors across the school and recruit key agents from the substructures.

### Communicate more effectively the system-level approach

The second recommendation focuses on introducing co-production into schools more effectively. This can be achieved through communicating clearly that this is a system-level approach which aims to involve students, staff, senior management and external stakeholders. In the framework this is highlighted in the ‘Intervention Readiness’ and ‘Group Development’ functions which rely on schools understanding the approach’s system-level aims. This was important as aligning the intervention too closely to ‘student voice’ and school councils had the unintended consequence of hindering recruitment and retention of RAG members in three ways.

First, as shown above, staff in school 1 were indifferent to the intervention with some not attending the co-production meetings as planned. It was believed that at least one of the Directors of Wellbeing didn’t attend meetings as he misrepresented the intervention as solely a mechanism to involve students.

(Director) emphasised the importance of the project from the aspect of listening to them. He said the pastoral team, they were adults and it has been a long time since they went to school, so they might not always understand the wellbeing issues they deal with. (Research Diary).

Secondly, in both schools there was a lack of effort put into recruiting external stakeholders such as parents and Governors. Study findings suggested that there was a complex relationship between schools and parents with parental engagement being difficult not just for this project but in other school business too. However, this cannot account for the lack of attempt to engage Governors, as they are already active members of the school community. A more fitting explanation appeared to be that staff prioritised student recruitment because they perceived engagement with the wider community as optional.

INT: So you talked about governors do you think we should’ve had governors on…

RES: Possibly yeah. Yeah, possibly governors would’ve been, would’ve been a good one. I mean I’m a governor of erm outside of school and I would’ve liked to have sort of, if my school runs something like this, I would’ve been nice to be involved (School 2, Staff 3).

Lastly, there was evidence that students within schools aligned the co-production project to school councils, which a few had negative experiences of:

RES: The last time I did it [school council], when I was in Year 10, 2016 and’17. So that was the problem, as we were coming up with all different event, I’m sorry, environmental ideas. For around the school, now and we also planned about recycling.

INT: Mmm.

RES: And re-using the er lost and found er school uniform. That was not discussed, it was discussed, but it wasn’t carried out. It was supposed to be carried out by the pupils, but there was no sort of staff observation. (School 2 Student 10).

These previous sections demonstrate that there would be value in separating this co-production intervention from school councils in Wales. This is especially important as the school council agenda engrains a separatist approach as they require schools to develop a structure of students with only one staff member [39]. Those in the school systems reverted to this understanding of involvement, showing a need to emphasis the involvement of actors throughout the school system. To achieve this, there was a need to alter the purpose of the ‘Group Development’ Function to include promoting the aims of co-production. This was considered most appropriate as stakeholders found this function was the least useful (see Additional file 1: Table S2) because RAG’s tended to already have members with good relationships. This allows the space in that co-production function to support stakeholders to understand the system level approach taken.

### Consistent involvement of SMT

The third recommendation focuses on working in a more consistent way with the SMT. This involved including three further functions where the SMT could check the diversity of RAGs, verify priorities raised, and assess the validity of solutions produced (see Fig. 2). In the initial framework, co-production was conceived as temporally sequential with all RAG functions preceding the SMT adoption function, where they accepted or not the mental health and wellbeing priorities. The two examples given below support why there was a need to change this.

The first example links to the SMT at school 2 raising at the plan adoption stage that they felt a priority on mental health was missing from their school plan.

RES4: One of the big things and I, I haven’t heard us mention that if I’m honest, I haven’t seen if I’ve read it in here is mental health. So we’re going to do a big thing on mental health.

RES1: No, this was a big thing in this, when the report was generated is myself and (sta3) said straight away they have, the pupils didn’t prioritise mental health. (School 2, SMT)

The SMT felt the RAG did not include ‘less engaged’ or ‘struggling’ students who would have prioritised mental health support. This led to stakeholders’ feeling there was a need for SMT to assess the diversity of RAGs before the groups start to prioritise issues.

A further example emphasised that involving SMT before adoption may have mitigated student expectations that all the priorities set by the RAG members would be accepted by the senior managers. It was clear throughout student interviews that they had a belief the school decision-makers would take on their ideas:

RES: I think they like will realise that these are things that can affect how school is for people so they’ll like want to change the school that they work in. (School 2, Student 4)

Actions to mitigate this were taken through reminding students that it was not feasible for all ideas to be taken on and reiterating to students the SMT adoption process. There was also a feeling that students involved in this co-production intervention, would now be aware of the complexities of school decision-making, and the need to balance targeting the right issues with feasibility and acceptability concerns. Ultimately though students had become deeply engaged in long term projects which did raise their expectations. Hence, there is also a moral imperative for the flow between RAG decision-making and SMT commitment to ensure RAG members are informed quickly if their ideas cannot be taken forward.

It is considered that the original understanding of power between the RAG structure and the SMT was one of a static, economic model where power could be transacted as a commodity [13] from the SMT, as school decision-makers, to the RAG. Whereas the changes proposed to the framework allow a more relational understanding of power where decisions are made by the flow of information between these two structures. Achieving this will involve integrating the three functions of ‘Diversity Check’, ‘Verifying Priorities’ and ‘Solution Validity’ into system activities already taking place such as SMT regular meetings.

### Involving wider stakeholders in problem-solving

The fourth recommendation of change was to include wider stakeholders such as health practitioners and researchers in RAGs, so they can support school stakeholders’ to decide on solutions. Who was involved in RAGs for the current study was directed by the previous co-production review. This recommendation change can be seen in the framework in the ‘Problem Solving’ function. The need for this change unfolded due to the following.

Difficulties in undertaking problem-solving decision-making were highlighted by the researcher and RAG participants throughout implementation data. Meeting observations in both schools showed that during problem-solving meetings the support needs of RAGs increased (see Additional file 1: Table S3). This was because RAGs found problem-solving cognitively demanding and often, they had a lack of knowledge to make informed choices on what solutions could best change the wellbeing issues in their school. For example, when asked to brainstorm different levels to tackle wellbeing priorities, staff and students both thought this was difficult:

RES: I found it good but some of the stuff like what will we do with the community, like if it’s in the school it’s school’s problem, if it’s, if something actually happens something very bad then it has to go to the community then. So we’re technically like sharing a bit.

INT: So did you find it easy to think of ways the community could help the school to change?

RES: Erm no. I couldn’t think of anything for the communities. (School 2, Student 8).

They also raised that students often had a lack of knowledge of the roles and responsibilities of staff members which were not linked to their teaching workload. This made it difficult for students to decide who should be responsible for actions. Observations also showed staff took the lead in action planning or worked hard to keep students involved in conversations. Some staff thought activities could be changed:

RES: I think if, if we had kind of a, a map. Err of, of the staff, to show who was employed at the school at different levels, I think that could have helped, because then they would have been able to say, “Actually that one doesn’t need to go to senior management”. “That, we could go straight to the Heads of Faculty with that one” (School 2, Staff 2).

To aid RAGs, the researcher decided to take a more proactive approach in both schools and gave some ideas of potential solutions that could support their priorities. However, when assessing the social validity of plans, issues were still raised because school stakeholders tended to raise solutions which could increase the knowledge of students but may not lead to behaviour change. Co-production may function more effectively through introducing wider suprasystem stakeholders to support solution generation and action planning [26]. These could include public health practitioners and researchers, and staff from other schools who may have examples of how their schools have targeted an issue. As part of this, RAGs could have cross-school problem-solving activity days with these wider stakeholders.

## Discussion

The study aimed to apply and test RAGs in secondary schools to optimise a co-production framework for developing public health interventions. This was in response to the lack of guidance or frameworks that can support the development of contextspecific interventions. The optimised framework (Fig. 2) is intended as a step-by-step guide to be used by researchers to support co-production with relevant stakeholders (for example, school students and staff). The study results have implications specifically for school-based co-production, and more generally for developing interventions, and for education policy and practice.

### Implications for school-based co-produced interventions

This study progresses knowledge about the use of RAGs to achieve co-production in school settings. Whilst evidence about how RAGs are received and implemented in school contexts has been accumulating [1, 12, 36], this study is novel in focusing on how to optimise the functions to guide the use of RAGs. The study identified four recommendations to do this. First, schools should assess the readiness of their baseline contexts to undertake co-production, and where necessary conduct preparatory work such as recruiting key agents, to ensure co-production is embedded extensively. Secondly, it is important to increase school stakeholders’ understanding of the system-level approach to ensure effort is put into recruiting and retaining all stakeholders. Thirdly, it is crucial to involve SMTs in decision-making throughout co-production to avoid RAGs setting priorities or solutions that the SMT will not adopt. Fourthly, problem-solving needs to include stakeholders from the wider health and research systems to support decision-making.

Taken together, these four recommendations complement other process evaluations of RAGs. For example, Warren et al. [37] also found one school RAG was less effective in implementing their actions partially due to a lack of wider school support. This resonates with the need to increase the readiness of schools to integrate co-production. However, the RAGs in Warren et al (36, 37) were part of multi-component interventions where co-produced activities were run alongside upskilling staff and students. Questions remain about whether RAGs should be used as the sole component or encompassed into multi-component interventions. This decision is influenced by intervention funders who have been reluctant to fund projects based purely on co-production [3]. It is hoped the accumulating knowledge about RAGs can go some way to developing confidence in this approach.

### Implications for developing public health interventions

The study progresses current complex intervention development and evaluation guidance [9, 18, 27, 34, 41] by providing a framework that can operationalise context-specific co-production. Whilst the importance of involving stakeholders is clearly articulated across these documents, public health intervention development has been dominated by co-production frameworks that develop standardised interventions [18, 41]. The current framework presents the functions necessary to operationalise an approach to co-production that has the potential to ensure that interventions can meet the needs of local contexts, possibly addressing commonly found implementation issues (e.g. [10, 11, 19, 32]). Further application and testing of this framework is needed with a focus on whether this approach can lead to better population-level outcomes. Further framework modifications could be made, and the addition or removal of functions for different health areas, settings, and/or populations should be explored.

This study also provides an important lesson about the balance between using academic and contextualised theories in intervention development [26]. Historically, intervention development has privileged academic theories [25], often omitting stakeholder theories or using stakeholder involvement to fill gaps where academic understanding is inadequate [26]. The present approach to co-production is ‘target population-centred’ [28] centring the views of stakeholders who will be intervention recipients. Conversely, the research found that this approach overprivileged school stakeholders’ theories hence the need to modify problem-solving to allow wider system stakeholders to articulate assumptions too. It is thought that these wider stakeholder groups could involve public health researchers and practitioners who can feed in information about academic theory during the problem-solving decision-making process. This strikes a balance between these positions because the academic theory is not imposed on schools (as previously done) but can be proposed for stakeholders to consider whether it fits their context or not. This aligns closer to the ‘partnership’ approach [28] where decision-making is equally shared by researchers and lay stakeholders. However, this can be challenging due to power imbalances and competing priorities [34, 35, 42] so the inclusion of additional stakeholders should form part of further process evaluations on this framework.

There is also a need to explore alignment between this context-specific approach and the wider framework for the development and evaluation of complex interventions [34]. Whilst there is recognition of the non-linear, iterative pathway through the research phases in the MRC guidance, this approach does not only blur the boundaries between the phases or revisit earlier aims in later phases, but it conflates the development, feasibility, and evaluation phases into one research iteration. This is more akin to action research (e.g. [7, 23, 35] where continual cycles of planning, acting and reflecting are used. These may be valuable for complex systems thinking as it allows interventions to be modified once it is understood how the system has initially responded to them. However, this pushes the boundaries of intervention contextualisation further than previously suggested. For example, Hawe et al. [16] accept we should standardise the functions of interventions, although this has been operationalised through presetting the health functions but allowing their form to fluctuate by context. It still remains uncertain whether action research which involves co-creating the health areas to target in individual settings will be accepted in the field of intervention research or deemed too ‘out of control’ [16] to be a viable option for how we develop and evaluation interventions.

### Implications for policy and practice

It could be explored whether the proposed co-production approach might have future use in school-based policy and more widely. Within the UK context, both the Scottish [33] and Welsh [39] governments have engaged in curriculum reforms since the start of this research. These reforms seek to centre efforts to improve the health and wellbeing of students. Co-production could complement these reforms through changing school systems to make them conducive to promoting student health. One such example of this is in Wales, where schools have a statutory requirement to co-produce action plans that can support the embedding of mental health and wellbeing [40]. However, schools’ capacity to do this is limited; policymakers will need to consider how they involve those external to the process to support it (i.e. researchers with knowledge on interventions) and how they can redistribute existing resources within the health and education system to co-production processes. This provides one example of a school-based approach, and policymakers and practitioners may look to utilise RAGs specifically, and co-production more broadly, across a range of substantive topics and settings.

### Limitations

There were two main limitations to this process evaluation. First is the dual role of the researcher as also the co-production facilitator, which divided the researcher’s attention between these two roles. This was mitigated by measures such as ensuring all observations were audio recorded so the researcher could return to research encounters within 48 h to verify all data was captured. Strategies were also used to limit Hawthorne effects by allowing RAG members to evaluate the project through surveys completed without the presence of the researcher. Second, was external validity, which was ameliorated through purposively sampling case study schools to support diversity in the characteristics of FSM entitlement, school size and geographical location, and grounding the findings in the wider literature on RAGs. Nonetheless, future co-production studies would benefit from larger sample sizes.

## Conclusion

This study progresses understanding of how to co-produce school-based health interventions. The proposed framework articulates the functions needed to enact co-production through RAGs. It also contributes to public health intervention development more broadly, as it moves beyond existing frameworks to provide a distinct approach for developing contextspecific interventions. Further research is required to apply and test the framework in different health areas, settings, and populations.

### Supplementary Information

**Additional file 1. Table 2: **Aggregated RAG members’ survey responses on their experience of co-production functions.

## Data Availability

The datasets generated and analysed during the current study are not publicly available to preserve participant anonymity.

## References

[1] Bell PB (2017). Promoting universal psychological well-being in an urban U.S. public school using a culture-specific, participatory action research approach to consultation. Int J School Educ Psychol.

[2] Bond L (2001). Building capacity for system-level change in schools: lessons from the gatehouse project. Health Educ Behav.

[3] Bonell C (2010). A pilot whole-school intervention to improve school ethos and reduce substance use. Health Educ.

[4] Bonell C (2012). Realist randomised controlled trials: a new approach to evaluating complex public health interventions. Soc Sci Med.

[5] Bonell C (2015). Initiating change locally in bullying and aggression through the school environment (INCLUSIVE): a pilot randomised controlled trial. Health Technol Assessment.

[6] Braun V, Clarke V (2019). Reflecting on reflexive thematic analysis. Qual Res Sport Exercise Health.

[7] Burns D (2021). The SAGE handbook of participatory research and inquiry.

[8] Clarke A (2021). Adolescent mental health: a systematic review on the effectiveness of school-based interventions.

[9] Craig P (2018). Taking account of context in population health intervention research: guidance for producers, users and funders of research.

[10] Durlak JA (2016). Programme implementation in social and emotional learning: basic issues and research findings. Camb J Educ.

[11] Evans R (2015). Implementation of a school-based social and emotional learning intervention: understanding diffusion processes within complex systems. Prev Sci.

[12] Fletcher A (2015). Involving young people in changing their school environment to make it safer: findings from a process evaluation in English secondary schools. Health Educ.

[13] Gallagher M (2008). Foucault, Power and Participation. Int J Child Rights.

[14] Gitlin LN (2013). Introducing a new intervention: an overview of research phases and common challenges. Am J Occup Ther.

[15] Hawe P (2000). Indicators to help with capacity building in health promotion.

[16] Hawe P (2004). Complex interventions: how “out of control” can a randomised controlled trial be?. BMJ.

[17] Hawe P (2009). Theorising interventions as events in systems. Am J Community Psychol.

[18] Hawkins J (2017). Development of a framework for the co-production and prototyping of public health interventions. BMC Public Health.

[19] Humphrey N, et al. Social and Emotional Aspects of Learning (SEAL) Programme in Secondary Schools. Manchester: Department for Education. 2010.

[20] Johnstone KM (2018). A meta-analysis of universal school-based prevention programs for anxiety and depression in children. Clin Child Fam Psychol Rev.

[21] Keshavarz N (2010). Schools as social complex adaptive systems: a new way to understand the challenges of introducing the health promoting schools concept. Soc Sci Med.

[22] Langford R (2014). The WHO Health Promoting School framework for improving the health and well-being of students and their academic achievement. Cochrane Database Syst Rev.

[23] Levy P (2023). Research design: quantitative, qualitative, mixed methods, arts and community-based participatory research approaches.

[24] Moore G (2015). Process evaluation of complex interventions: medical Research Council guidance. BMJ.

[25] Moore G, Evans R (2017). What theory, for whom and in which context? Reflections on the application of theory in the development and evaluation of complex population health interventions. SSM Popul Health.

[26] Moore G (2019). From complex social interventions to interventions in complex social systems: future directions and unresolved questions for intervention development and evaluation. Evaluation.

[27] O’Cathain A (2019). Guidance on how to develop complex interventions to improve health and healthcare. BMJ Open.

[28] O’Cathain A (2019). Taxonomy of approaches to developing interventions to improve health: a systematic methods overview. Pilot Feasibility Stud.

[29] Perez Jolles M (2019). Core functions and forms of complex health interventions: a patient-centered medical home illustration. J Gen Intern Med.

[30] Reed H (2021). Co-production as an emerging methodology for developing school-based health interventions with students aged 11–16: systematic review of intervention types, theories and processes and thematic synthesis of stakeholders’ experiences. Prev Sci.

[31] Rutter H (2017). The need for a complex systems model of evidence for public health. The Lancet.

[32] Sadjadi M (2021). Barriers and facilitators to the implementation of Health-Promoting School programmes targeting bullying and violence: a systematic review. Health Educ Res.

[33] Scottish Government. 2018. *Curriculum for Excellence*. Available at: https://education.gov.scot/Documents/All-experiencesoutcomes18.pdf [Accessed: 28/11/22].

[34] Skivington K, et al. Framework for the development and evaluation of complex interventions: gap analysis, workshop and consultation-informed update. 2021; 25:57. 10.3310/hta25570. 10.3310/hta25570 PMC7614019 34590577

[35] Stokols D (2006). Toward a science of transdisciplinary action research. Am J Community Psychol.

[36] Warren E (2019). Action groups as a participative strategy for leading whole-school health promotion: results on implementation from the INCLUSIVE trial in English secondary schools. Br Edu Res J.

[37] Warren E (2020). Using qualitative research to explore intervention mechanisms: findings from the trial of the learning together whole-school health intervention. Trials.

[38] Wells M (2012). Intervention description is not enough. Trials.

[39] Welsh Government. 2021a. The Curriculum and Assessment (Wales) Act: explanatory memorandum.

[40] Welsh Government (2021). Framework on embedding a whole-school approach to emotional and mental well-being.

[41] Wight D (2016). Six steps in quality intervention development (6SQuID). J Epidemiol Community Health.

[42] Williams O (2020). Lost in the shadows: reflections on the dark side of co-production. Health Res Policy Syst.

