# Supplementary material for: Optimising a co-production framework for developing public health interventions: application and testing of school-based Research Action Groups
Source: Health Res Policy Syst. 2023 Dec 14;21:133. doi: 10.1186/s12961-023-01086-3 (PMC10720185; doi:10.1186/s12961-023-01086-3)
Supplement: Supplementary file 1 — Additional file 1. Table 2: Aggregated RAG members’ survey responses on their experience of co-production functions. [file 12961_2023_1086_MOESM1_ESM.docx]

*Supplementary Materials*

*Table 2: Survey Reponses*

| Functions | | Responses | School 1  N (%) | School 2  N (%) |
| --- | --- | --- | --- | --- |
| RECRUITMENT | 1. 1 Did the action group include students from a range of different backgrounds? | Very good range | 9 (81.8) | 6 (40.0) |
|  |  | Quite a good range | 2 (18.2) | 8 (53.3) |
|  |  | Not a good range | 0 (0.0) | 1 (6.7) |
|  |  | Not a good range at all | 0 (0.0) | 0 (0.0) |
|  | 1. 2 Did the action group include a range of different staff from across the school | Very good range | 2 (18.2) | 8 (53.3) |
|  |  | Quite a good range | 8 (72.7) | 7 (46.7) |
|  |  | Not a good range | 1 (9.1) | 0 (0.0) |
|  |  | Not a good range at all | 0 (0.0) | 0 (0.0) |
| GROUP DEVELOPMENT | 1. 3 Were the group development activities useful in helping you understand how the group would function? | Very useful | 4 (36.4) | 5 (33.3) |
|  |  | Quite useful | 7 (63.6) | 8 (53.3) |
|  |  | Not useful | 0 (0.0) | 2 (13.3) |
|  |  | Not at all useful | 0 (0.0) | 0 (0.0) |
|  | 1. 4 Were the group development activities useful in helping you build a relationship with other members? | Very useful | 2 (18.2) | 7 (46.7) |
|  |  | Quite useful | 7 (63.7) | 5 (33.3) |
|  |  | Not useful | 1 (9.1) | 3 (20.0) |
|  |  | Not at all useful | 0 (0.0) | 0 (0.0) |
| PROBLEM SETTING | 1. 5 Were the photography themes useful in helping the action group understand wellbeing in the school? | Very useful | 9 (81.8) | 11 (78.6) |
|  |  | Quite useful | 2 (18.2) | 3 (21.4) |
|  |  | Not useful | 0 (0.0) | 0 (0.0) |
|  |  | Not at all useful | 0 (0.0) | 0 (0.0) |
|  | 1. 6 Were the statistics useful in helping the action group understand wellbeing in the school? | Very useful | 7 (63.6) | 7 (46.7) |
|  |  | Quite useful | 4 (36.4) | 7 (46.7) |
|  |  | Not useful | 0 (0.0) | 1 (6.7) |
|  |  | Not at all useful | 0 (0.0) | 0 (0.0) |
|  | 7 Did the prioritisation task help the action group decide on relevant wellbeing topics to tackle? | Very useful | 7 (70.0) | 11 (73.3) |
|  |  | Quite useful | 3 (30.0) | 3 (20.0) |
|  |  | Not useful | 0 (0.0) | 1 (6.7) |
|  |  | Not at all useful | 0 (0.0) | 0 (0.0) |
| PROBLEM SOLVING | 8 Did the brainstorming task help the action group decide on relevant solutions? | Very useful | 7 (77.8) | 7 (46.7) |
|  |  | Quite useful | 1 (11.1) | 6 (40.0) |
|  |  | Not useful | 1 (11.1) | 2 (13.3) |
|  |  | Not at all useful | 0 (0.0) | 0 (0.0) |
|  | 9 Did the last meetings help to finalise the action plan? | Very useful | 3 (37.5) | 10 (66.7) |
|  |  | Quite useful | 5 (62.5) | 5 (33.3) |
|  |  | Not useful | 0 (0.0) | 0 (0.0) |
|  |  | Not at all useful | 0 (0.0) | 0 (0.0) |
| SHARED DECISION-MAKING | 10 Did all action members have their say throughout the process? | Yes | 11 (100.0) | 12 (80.0) |
|  |  | No | 0 (0.0) | 2 (13.3) |
|  |  | Not Sure | 0 (0.0) | 1 (6.7) |
|  | 11 Overall, do you think this project was a good way to ensure that students and staff contribute to decision-making at this school? | Yes | 11 (100.0) | 14 (93.3) |
|  |  | No | 0 (0.0) | 0 (0.0) |
|  |  | Not Sure | 0 (0.0) | 1 (6.7) |
| EXTERNAL SUPPORT | 12 Was the researcher useful in ensuring that all action group members could have their say? | Very useful | 10 (90.9) | 13 (86.6) |
|  |  | Quite useful | 1 (9.1) | 1 (6.7) |
|  |  | Not useful | 0 (0.0) | 1 (6.7) |
|  |  | Not at all useful | 0 (0.0) | 0 (0.0) |
|  | 1. 13 Was the researcher useful in supporting the overall process? | Very useful | 9 (81.8) | 12 (80.0) |
|  |  | Quite useful | 2 (18.1) | 3 (20.0) |
|  |  | Not useful | 0 (0.0) | 0 (0.0) |
|  |  | Not at all useful | 0 (0.0) | 0 (0.0) |

Table 3: Mean observer ratings of RAG understanding, support needed, engagement and activity completion.

| School 1 | | | | |
| --- | --- | --- | --- | --- |
| Activity Meetings | Understanding | Support | Engagement | Completion |
| Problem Setting (meeting 1) | 1.67 | 0.33 | 1.67 | 2.00 |
| Problem Setting (meeting 2) | 2.00 | 0.50 | 2.00 | 1.50 |
| Problem Solving (meeting 3) | 1.50 | 1.00 | 1.50 | 1.00 |
| Problem Solving (meeting 4) | 2.00 | 2.00 | 1.00 | 1.00 |
| School 2 | | | | |
| Activity Meetings | Understanding | Support | Engagement | Completion |
| Problem Setting (meeting 1) | 1.67 | 0.33 | 1.33 | 2.00 |
| Problem Setting (meeting 2) | 1.50 | 0.50 | 1.33 | 1.50 |
| Problem Solving (meeting 3) | 1.50 | 1.50 | 1.00 | 1.00 |
| Problem Solving (meeting 4) | 2.00 | 2.00 | 1.00 | 2.00 |

^a^ Items assessed by researcher using a three-point Likert scale.

*Note, for some of the activities the larger RAG groups were split into smaller groups to undertake tasks* therefore mean ratings are used.

Group observations were structured around four criteria: Understanding - Whether members understood the aims of the group meetings; Support - Whether the researcher needed to facilitate none, part or all of meetings to achieve the aims; Engagement - Whether the group remained focused on the aims; Completion - Whether the group completed all aims.
